# Geospatial Variation in Rotavirus Vaccination in Infants, United States, 2010–2017

**DOI:** 10.3201/eid2510.190874

**Published:** 2019-10

**Authors:** Mary A.M. Rogers, Catherine Kim, Annika M. Hofstetter

**Affiliations:** University of Michigan, Ann Arbor, Michigan, USA (M.A.M. Rogers, C. Kim);; University of Washington and Seattle Children’s Research Institute, Seattle, Washington, USA (A.M. Hofstetter)

**Keywords:** Rotavirus, vaccines, infant, United States, public health surveillance, viruses

## Abstract

We evaluated rotavirus vaccination rates in the United States by using records from a nationwide health database. From data on 519,697 infants, we found 68.6% received the entire rotavirus vaccine series. We noted pockets of undervaccination in many states, particularly in the Northeast and in some western states.

Vaccination coverage in the United States frequently is evaluated with telephone and mailed surveys (*1*). However, telephone response rates have declined over the past 2 decades (*2*) and parents who choose not to vaccinate their children might be less likely to participate in surveys (*3*). 

The Advisory Committee on Immunization Practices (ACIP) recommends routine vaccination among US infants to prevent rotavirus infection, the most common cause of gastroenteritis in children worldwide (*4*). We designed a study to evaluate rotavirus vaccination rates using nationwide health insurance records.

We conducted a longitudinal study of rotavirus vaccination rates during January 1, 2010–June 30, 2017. We obtained deidentified data from Clinformatics Data Mart (Optum, https://www.optum.com), an integrated database containing demographic, service type (inpatient and outpatient), medication, and laboratory data for ≈77.8 million privately insured persons of all ages across 50 states. We included data on infants (<1 year of age) with continuous health insurance enrollment from birth to 1 year of age and an available residential ZIP code. We determined completion of the rotavirus vaccine series by using Current Procedural Terminology (CPT) codes and data on vaccine administration, including vaccine type, date, and location. Vaccine completion requires 2 doses of monovalent Rotarix (GlaxoSmithKline, https://www.gsk.com; CPT 90681) or 3 doses of pentavalent RotaTeq (Merck and Company, https://www.merck.com; CPT 90680). To evaluate geographic variation, we used the first 3 digits of residential ZIP codes and excluded areas with <20 infants to provide stability of the estimates. The study was reviewed and deemed exempt by the institutional review board of the University of Michigan.

We identified 526,376 infants with continuous health insurance for >1 year during 2010–2017. We excluded 5,708 (1.1%) with no known residential ZIP code and 971 (0.2%) from areas with <20 infants. Our final cohort contained 519,697 eligible infants; 99.8% had no copayment for vaccine administrations. The number of infants in each 3-digit ZIP code area was 20–9,426 (median 223; interquartile range 85–682).

In our cohort, 68.6% (95% CI 68.5%–68.8%) of infants completed the rotavirus vaccine series; 15.9% completed only part of the series, and 15.5% received no rotavirus vaccine. Of infants completing the vaccine series, 79% received RotaTeq, 19% received Rotarix, and 2% received both. The mean interval between vaccine doses was 64.9 days. 

Rotavirus vaccination rates were higher in eastern states, although some states in the Northeast had low proportions of vaccination (Figure). Alaska had considerably lower vaccination rates, ranging from 17% to 28%. Series completion was lowest in northeastern Wyoming at 9% (95% CI 4%–17%) and highest (>80%) in upstate New York, several areas of Pennsylvania, and 1 suburb of San Francisco, California.

**Figure:**
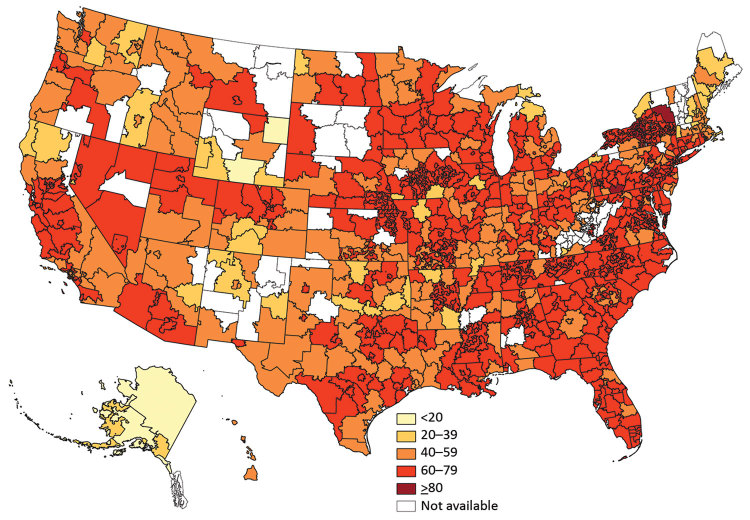
Percentage of infants (<1 year of age) covered by private health insurance who completed the rotavirus vaccination series in the United States, 2010–2017.

Rotavirus vaccination coverage varied considerably across the United States, with pockets of undervaccination in many states. National Immunization Survey (NIS) data show ≈59.2% of infants completed the rotavirus vaccine series in 2010 and ≈73.2% completed the series in 2017 (*1*,*5*). Our overall estimate was 68.6% during 2010–2017 among privately insured infants. However, we note several differences in these samples. NIS used a nationwide sample of 15,333 children in 2017, whereas our study used 34× that number (519,697), giving us an opportunity to assess vaccination rates in local areas. NIS weights for nonparticipation but could underestimate coverage rates in less densely populated areas (*6*). Our use of deidentified data rather than telephone surveys might provide an opportunity to include vaccine-hesitant populations (*3*). However, our sample does not shed light on vaccination rates in children covered under Medicaid or the Children’s Health Insurance Program or those with no insurance coverage.

Parents’ decision to vaccinate their children involves a complex interplay between advice from family and friends; school and institutional mandates; experience with healthcare professionals; personal beliefs; and social impacts, including media coverage, access, and transportation issues (*7*). The geographic variation in vaccination rates we found might reflect some of these determinants. For instance, low coverage in remote areas may reflect an inability to travel to providers; lower overall vaccination rates have been found in children living in rural areas (*1*,*8*). Because 99.8% of our cohort did not have a copayment, we do not believe there was financial disincentive, but other financial obstacles could exist. Previous studies suggest rotavirus vaccination is lower among persons with public or no insurance (*9*). Therefore, vaccination rates might be <68% in geographic regions with a high number of uninsured or underinsured children.

The 15.9% of infants in our cohort who did not complete the vaccination series could reflect an inability to meet the start- and end-date requirements. Rotavirus vaccination has an exceptionally narrow window of administration; ACIP recommends the first dose at <15 weeks of age and conclusion of all doses before 8 months of age. 

In summary, we found considerable geographic variation in rotavirus vaccination rates in the United States. We recommend additional efforts at the local and county levels to address pockets of rotavirus undervaccination.
